# *Perilimnastes dongchauensis* (tribe Sonerileae, Melastomataceae), a new species from Quang Trị province, Vietnam

**DOI:** 10.3897/phytokeys.273.177749

**Published:** 2026-04-15

**Authors:** Cuong Huu Nguyen, Nam Van Duong, Doi The Bui, Hung The Tran, Hai Thanh Bach, Quyet Thanh Phan, Vu Khanh Le, Duong Tuan Nguyen, Maxim S. Nuraliev, Leonid V. Averyanov, Che Wei Lin

**Affiliations:** 1 Vietnam National University of Forestry, Xuan Mai, Hanoi, Vietnam Biological Faculty, M.V. Lomonosov Moscow State University Moscow Russia; 2 Center for Experiment and Technology Development, Vietnam National University of Forestry – Gia Lai Campus, Hoi Phu, Gia Lai, Vietnam Herbarium of Taiwan Forestry Research Institute, Taiwan Forestry Research Institute Taipei Taiwan; 3 Quang Binh University, Ly Thuong Kiet, Dong Hoi, Quang Tri, Vietnam Quang Binh University Dong Hoi Vietnam; 4 Dong Chau – Khe Nuoc Trong Nature Reserve, Kim Ngan, Quang Tri, Vietnam Komarov Botanical Institute of the Russian Academy of Sciences St. Petersburg Russia; 5 Bac Giang Agriculture and Forestry University, Viet Yen, Bac Ninh, Vietnam Vietnam National University of Forestry Xuan Mai Vietnam; 6 Department of Higher Plants, Biological Faculty, M.V. Lomonosov Moscow State University, 1, 12, Leninskie Gory, 119234 Moscow, Russia Center for Experiment and Technology Development, Vietnam National University of Forestry – Gia Lai Campus Gia Lai Vietnam; 7 Komarov Botanical Institute of the Russian Academy of Sciences, Prof. Popov str., 2, St. Petersburg, 197022, Russia Dong Chau – Khe Nuoc Trong Nature Reserve Kim Ngan Vietnam; 8 Herbarium of Taiwan Forestry Research Institute, Taiwan Forestry Research Institute, No. 53, Nan–Hai Road, Taipei 100, Taiwan Bac Giang Agriculture and Forestry University Viet Yen Vietnam

**Keywords:** Biodiversity, endemics, plant taxonomy, *
Perilimnastes
*, Quang Tri province

## Abstract

*Perilimnastes dongchauensis* (Melastomataceae), apparently endemic to Quang Tri province, Central Vietnam, is described here as a new species. It closely resembles *P. suberalata* in having elliptic-lanceolate leaf blades, few-flowered inflorescences, 4-merous flowers, and similar anther morphology. However, it differs in several key characteristics, with stems and petioles densely setose (vs. glabrous), leaves equal or subequal in a pair (vs. unequal, rarely subequal) and adaxial surface setose (vs. glabrous), hypanthia setose (vs. glabrous), calyx lobes narrowly triangular (vs. linear), and petals much larger, measuring 17–20 × 13–18 mm (vs. ca. 6 × 3.5 mm).

## Introduction

Vietnam is part of the Indo-Burma biodiversity hotspot, one of 36 such hotspots recognized globally (Conservation International 2026). Its vascular flora comprises an estimated 12,000 species, with national endemism reaching about 30% and up to 50% in the north (Regalado et al. 2005). Despite long-standing anthropogenic impacts on ecosystems such as the lowland broad-leaved rainforest (Wikramanayake et al. 2002), new species continue to be discovered annually, including in degraded forests that still harbor potentially threatened taxa (Cochard et al. 2017).

Over the past decade, taxonomic research on Melastomataceae in Vietnam has intensified, receiving increased attention and leading to a steady stream of new discoveries (Dang et al. 2016; Pham et al. 2017; Dai et al. 2022, 2023; Lin et al. 2022; Liu et al. 2022, 2024; Nguyen et al. 2022, 2024a, 2024b, 2025; Phung et al. 2025; Tagane 2025). *Perilimnastes* Ridley (Ridley 1918) is a genus of Sonerileae, and recent revisions by Liu et al. (2024) recognized 15 new combinations, raising the total to 22 species, 10 of which are endemic to Vietnam. In this study, we describe a newly discovered, locally endemic species of *Perilimnastes* from Central Vietnam. This finding not only deepens our understanding of species diversity within the Vietnamese Melastomataceae but also contributes to broader conservation efforts by highlighting the potential for nature-based tourism as a means of alleviating environmental pressures associated with land-use change.

### Key to the species of *Perilimnastes* from Indochina and China

**Table:** 

1	Raphides present, appearing as whitish oblong spots on dried leaf surfaces	**2**
–	Raphides absent	**10**
2	Flowers solitary	***P. uniflora***
–	Flowers umbellate, rarely solitary or many-flowered	**3**
3	Inflorescences sessile or subsessile	**4**
–	Inflorescences pedunculate, peduncles 1–3.5 cm long	**7**
4	Leaf blades narrowly elliptic to obovate-lanceolate	**5**
–	Leaf blades broadly elliptic or elliptic	**6**
5	Hypanthia with bristles; sepals narrowly elliptic-oblong; anthers purplish	***P. guillauminii***
–	Hypanthia strigose or tuberculate-based strigose; sepals very narrowly triangular to linear-triangular; anthers yellowish	***P. dongchauensis***
6	Stems and petioles with appressed gland-tipped villous hairs, without stellate pubescence	***P. banaensis***
–	Stems and petioles with stellate pubescence	***P. setipetiola***
7	Stems with crooked multiseriate hairs	**8**
–	Stems with straight multiseriate hairs	**9**
8	Leaf blades 4.8–14 × 1.1–2.7 cm; peduncles with appressed hairs	***P. elegans***
–	Leaf blades 5–13 × 1.5–4 cm; peduncles with spreading hairs	***P. deltoidea***
9	Stems pubescent or retrorse-hirsute; leaf blades 7–18 × (2–)3–8.5 cm	***P. ovalifolia***
–	Stems setose; leaf blades 5–8 × 2.5–4 cm	***P. ternata***
10	Leaf bases broadly cordate	***P. sessilifolia***
–	Leaf bases cuneate to acuminate	**11**
11	Mature stems and leaves with bristles	***P. melastomatoides***
–	Mature stems and leaves glabrous	**12**
12	Leaves unequal in a pair	***P. suberalata***
–	Leaves equal or subequal	**13**
13	Leaf blades 10–20 × 3–8 cm; inflorescences subtended by an involucre of several bracts (often 4)	***P. setotheca***
–	Leaf blades 2.8–10(–14) × 0.6–2.4(–4.2) cm; inflorescences subtended by a pair of small leaf/bracts	**14**
14	Calyx lobes 4–8	***P. multisepala***
–	Calyx lobes 4	**15**
15	Plants to 15 cm tall; inflorescences 1-flowered	***P. nana***
–	Plants to 80–100 cm tall; inflorescences 1–4-flowered	***P. stenophylla***

## Taxonomic treatment

### Perilimnastes dongchauensis

Taxon classificationPlantaeMyrtalesMelastomataceae

C.H.Nguyen, D.V.Nam & C.W.Lin sp. nov.

5F8DB2B4-A73A-559E-9552-699968637B3D

urn:lsid:ipni.org:names:77378627-1

Figs 1, 2, 3

#### Type.

Vietnam • Quang Tri province, Kim Ngan commune, primary and old secondary evergreen broad-leaved forest, at elevations of 100–200 m a.s.l., along streams and on stream valley slopes in wet rocky places with soil rich in humus, around point 16°59'25"N, 106°43'00"E, locally common, 8 July 2025, *Nguyen Huu Cuong, Duong Van Nam, Tran The Hung, Phan Thanh Quyet, Le Khanh Vu NHC 250708040* (holotype: VNF!; isotypes: VNF!, LE!).

**Figure 1.**
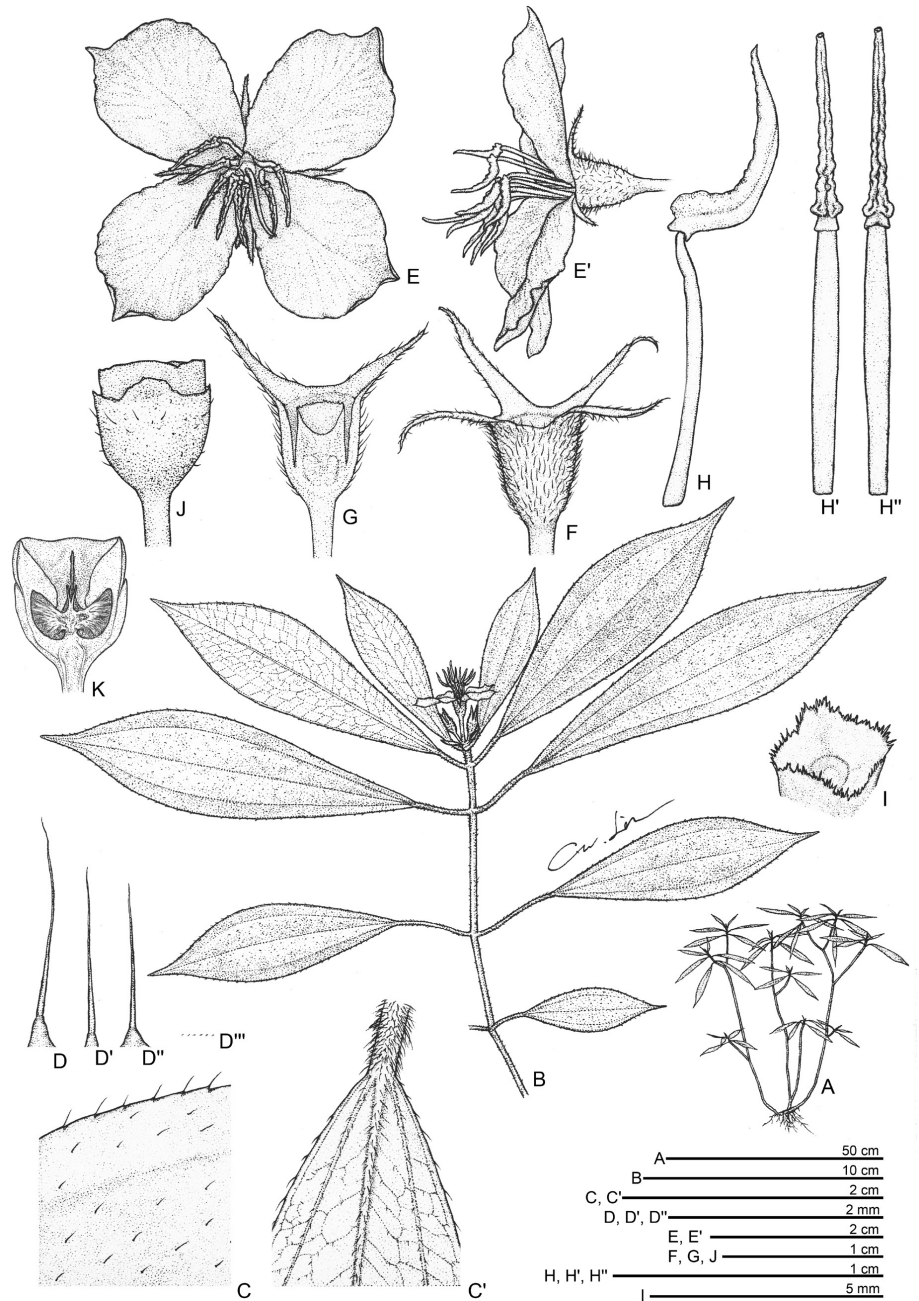
*Perilimnastes dongchauensis*. **A**. Habit; **B**. Flowering branch; **C**, **C’**. Portion of leaf, adaxial and abaxial surfaces; **D**, **D’, D’’**. Strigose and tuberculate-base strigose; **D’’’**. Glandular trichomes; **E**, **E’**. Flower, front and lateral views; **F**. Hypanthium; **G**. Longitudinal section of ovary; **H, H’, H’’**. Stamens, side and ventral views; **I**. Ovary Crown; **J**. Capsule; **K**. Longitudinal section of capsule. All drawn from the holotype, *NHC 250708040* by Che Wei Lin.

**Figure 2.**
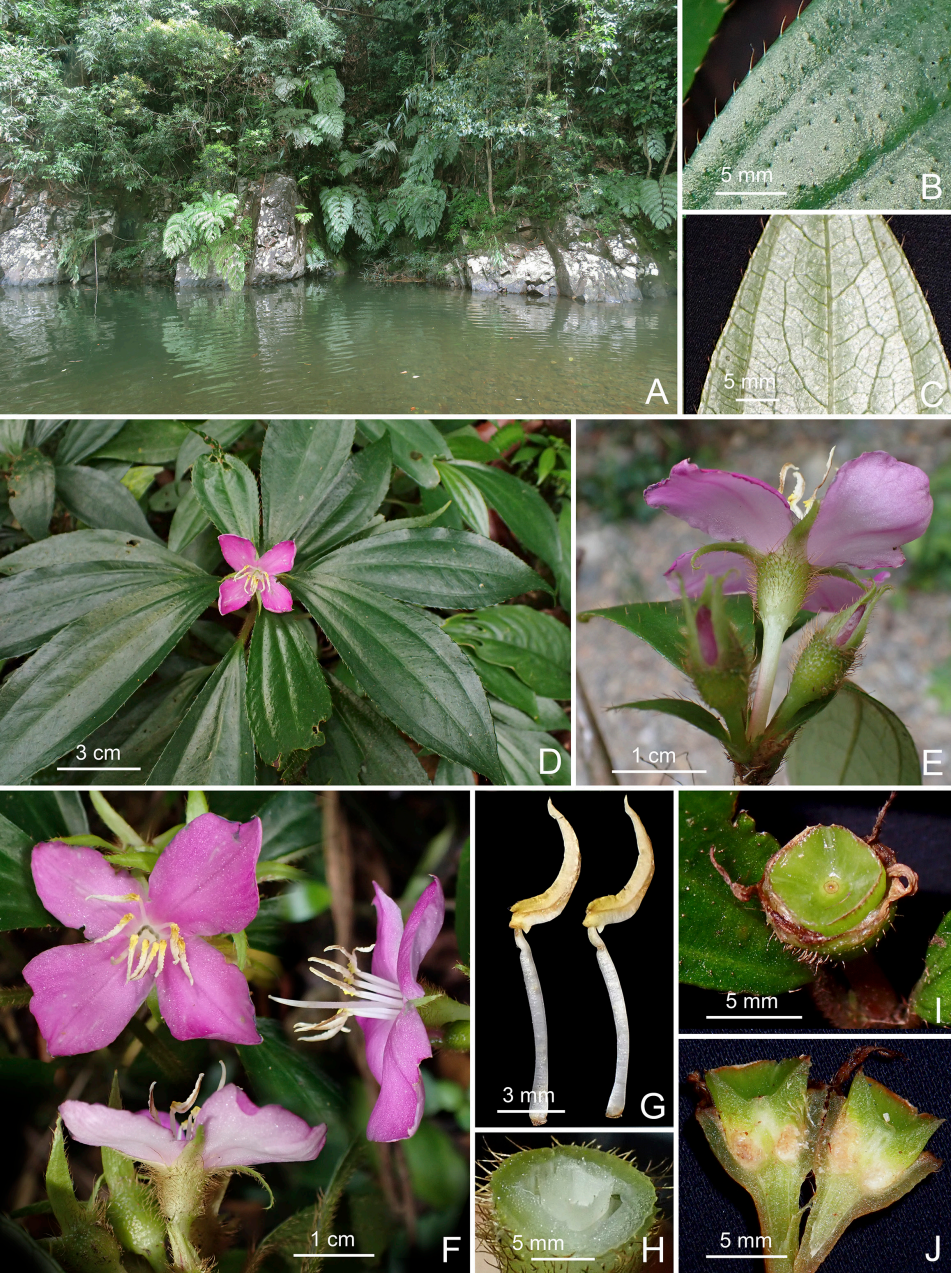
*Perilimnastes dongchauensis*. **A**. Habitat, the new species grows on shaded rocks above the waterline along streams in humid evergreen forest; **B**. Portion of leaf, adaxial surface; **C**. Portion of leaf, abaxial surface; **D**. Flowering branch; **E**. Inflorescence, also showing leaf-like bracts; **F**. Flowers, front and lateral views; **G**. Stamens, side view; **H**. Ovary Crown; **I**. Capsule; **J**. Longitudinal section of capsule. All photos by Cuong Huu Nguyen from specimens used for preparation of the holotype and isotypes.

**Figure 3.**
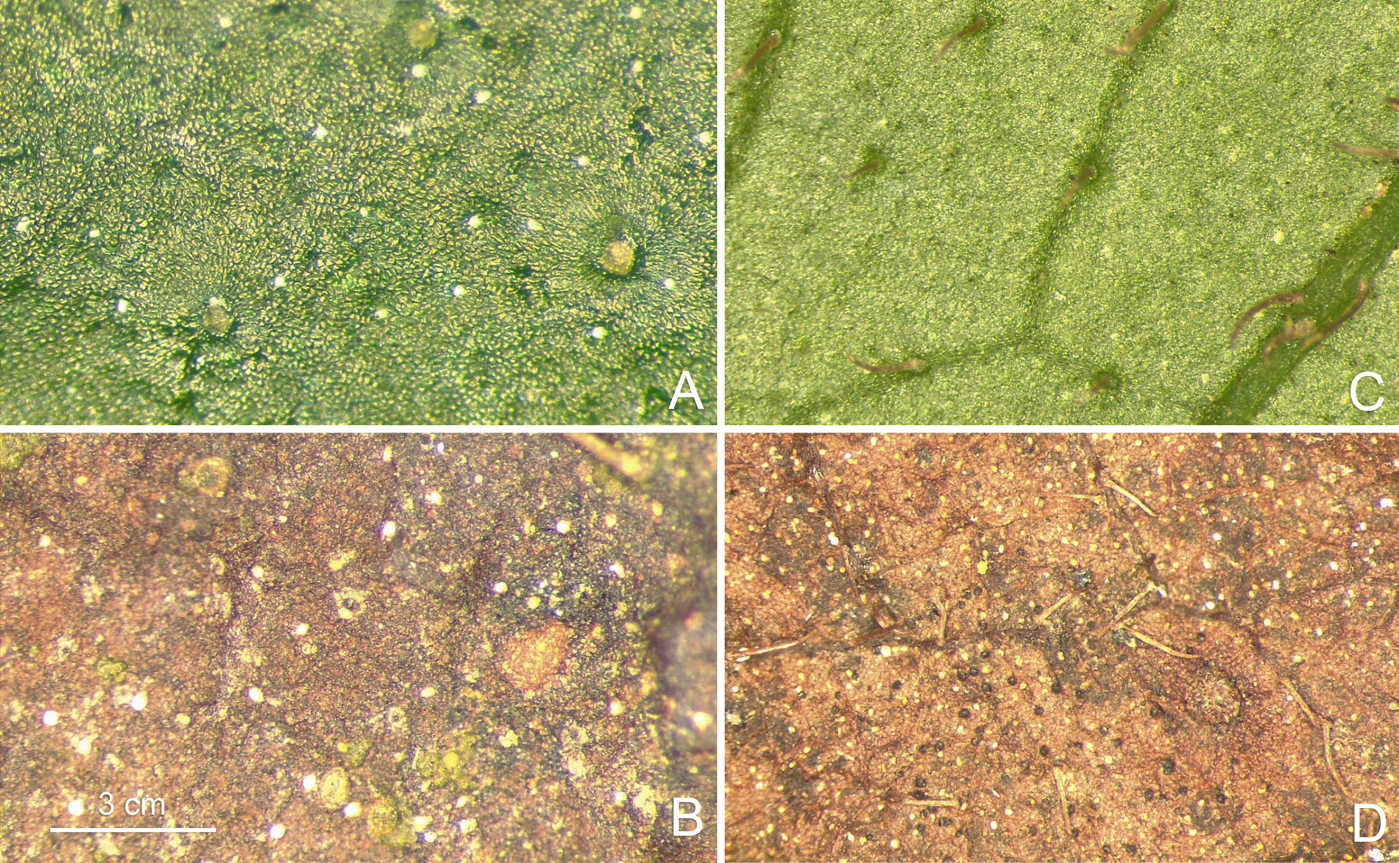
*Perilimnastes dongchauensis*. Raphides (as white spots) on adaxial and abaxial leaf surfaces at living plant and holotype specimen. **A, B**. Adaxial surface; **C, D**. Abaxial surface. All photos by Cuong Huu Nguyen from specimens used for preparation of the holotype and isotypes.

#### Diagnosis.

*Perilimnastes dongchauensis* resembles *P. suberalata* (C.Hansen) Ying Liu (Hansen 1990; Liu et al. 2024) in having elliptic-lanceolate leaf blades, few-flowered inflorescences, 4-merous flowers, and similar anther morphology. However, it differs in several key characters, with stems and petioles densely setose (vs. glabrous), leaves equal or subequal in a pair (vs. unequal, rarely subequal) and adaxial surface setose (vs. glabrous), hypanthia setose (vs. glabrous), calyx lobes narrowly triangular (vs. linear), and petals much larger, measuring 20 × 18 mm (vs. 6 × 3.5 mm).

#### Description.

Lithophytic, suberect to ascending, multi-branched shrublets to 80 cm or taller, with raphides in many parts. Stems olive green to brownish-red, becoming woody and pale grayish-brown with age; obtusely quadrangular in cross-section, internodes (0.3–)1–3(–7) cm long, 2–3.5 mm thick in distal branches, up to 7 mm or more in the lower stems, minute glandular trichomes mixed with moderately to densely golden brown strigose (1–3 mm long), glabrescent. Leaves simple, decussate and held horizontally, equal to subequal in a pair; petiole olive green to brownish-red, 8–50 mm long, oblate in cross-section, grooved above, minute glandular trichomes mixed with densely golden brown strigose; leaf blade oblanceolate to obovate-lanceolate, 5–16 × 1.5–5 cm, chartaceous, adaxial surface dark green, sparsely golden brown strigose (*ca*. 1.5 mm long) between veins; abaxial surface pale green, minute glandular trichomes mixed with sparsely golden brown strigose along veins; base cuneate to attenuate, apex attenuate-acuminate to acuminate-caudate, margin inconspicuous coarsely serrate, each tooth tipped by a strigose hair; venation acrodromous, with 3 primary veins originating from the base, slightly prominent to flat on the adaxial surface and prominent on the abaxial surface, secondary and tertiary veins conspicuous on the abaxial surface. Inflorescences terminal, umbellate cymes with 3–6 flowers; peduncles sessile or subsessile, bearing a single pair of leaf-like bracts; bracts 6–15 × 2–5 mm. Flowers 4-merous; pedicel greenish white tinged pink towards base, terete, 10–15 mm long in flower, up to 18 mm long in fruit, glabrous. Hypanthium funnel-shaped, greenish white; 5–7 mm long, 5–6 mm in diameter, minute brown glands mixed with moderately to densely golden brown strigose or tuberculate-base strigose. Sepals 4, very narrowly triangular to linear-triangular, greenish white tinged green towards apex, 10–12 mm long, 1.2–1.5 mm wide at the middle and *ca*. 3 mm wide at the base, adaxially minute glandular trichomes mixed with golden brown strigose, margins subentire with rows of strigose indumentum. Petals 4, bright pink adaxially, pinkish white abaxially, broadly obovate-oblong, slightly oblique, 17–20 × 13–18 mm, glabrous. Stamens 8, isomorphic, glabrous, filamentous, all anthers grouped together above the stigma; filaments subterete, *ca*. 10 mm long, white; anthers subulate, strongly incurved, 6.5–8 mm long (appendages included), creamy white tinged with yellow towards the base; ventrally with a pair of bright yellow, tuberculate lobes at the base of the anther sacs; connective dorsally forming a *ca*. 0.3 mm long spur and ventrally bearing a truncate appendage, sometimes retuse at the middle, nearly forked into two triangular lobes; apically with one pore. Ovary *ca*. 1/3 as long as hypanthium (crown excluded), 1.5–2 mm long, 4-locular; ovary crown wedge-like, 4-lobed, margin denticulate, 1.2–1.5 mm high; ovules numerous; ovary adnate to hypanthium for 1–2 mm. Style filiform, white, 1.8–2.2 cm long; stigma obscurely capitate. Capsule cup-shaped, 6.5–8 × 5–7 mm, quadrangular; hypanthium 8-ribbed; crown enlarged enclosing an obpyramidal space; placental column unbeaked, 4-horned; placenta thread-like. Seeds numerous, oblong-ovoid to narrowly triangular-oblongoid, (0.3–) 0.6–0.8 mm long, 0.25–0.35 mm thick, brown.

#### Distribution.

*Perilimnastes dongchauensis* is currently known only from Quang Tri province, Central Vietnam, including Dong Chau-Khe Nuoc Trong Nature Reserve and Phong Nha-Ke Bang National Park.

#### Habitat.

*Perilimnastes dongchauensis* grows in primary and old secondary lowland, evergreen, broad-leaved forests dominated by *Amesiodendron chinense* (Merrill) Hu, *Dillenia heterosepala* Fin. & Gagnep., *Syzygium attopeuense* (Gagnep.) Merr. & LMPery, and *Elaeocarpus hainanensis* Oliver, *Impatiens claviger* J.D.Hooker, along streams and stream valley slopes in wet rocky places with soil rich in humus at elevations of 100–750 m a.s.l., locally common.

#### Phenology.

Flowering from June to November, fruiting from July to December.

#### Etymology.

The species is named after its type locality, Dong Chau-Khe Nuoc Trong Nature Reserve.

#### Vietnamese names.

Me đá động châu.

#### Preliminary IUCN conservation status.

The species was found in some locations as a common plant, able to grow well in secondary and primary forests in the territory that is currently officially protected. According to the criteria and terms of the Red List IUCN (2024) we preliminarily estimate the conservation status of *P. dongchauensis* as being of least concern (LC).

#### Note.

Within the Sino-Vietnamese region, *P. dongchauensis* shows some resemblance to *P. elegans* (Hai L. Chen, Yan Liu & Ying Liu) Ying Liu (Chen et al. 2021; Liu et al. 2024), sharing a hairy stem, oblanceolate 3-veined, leaves setose on the adaxial surface, and an umbellate inflorescence and flowers with pink petals. However, it can be readily distinguished from *P. elegans* by its setose stems (vs. crooked hairs), consistently 4-merous flowers (vs. usually 3-merous), much shorter peduncles (0.3–0.6 mm vs. 15–27 mm), setose hypanthia (vs. glandular), whitish yellow anthers (vs. purple), and a truncated, ventral connective appendage (vs. absent).

#### Additional material studied (paratypes).

Vietnam • Quang Tri province: Kim Ngan commune, old secondary evergreen broad-leaved forest, at elevation *ca*.150 m a.s.l., along streams, around point 16°59'00"N, 106°43'00"E, locally common, 8 October 2025, *Cao Xuan Tuan, Bach Thanh Hai, DCKNT - NR 251008002* (VNF); • Phong Nha-Ke Bang National Park, *ca*. 700 m a.s.l., around point 17°28'21.5"N, 106°22'44.6"E, 27 November 2025, *T.C.Hsu16510* (SGN, TAIF).

## Supplementary Material

XML Treatment for Perilimnastes dongchauensis

## Additional information

### Artificial Intelligence (AI) use

The authors accept full responsibility for the content of the manuscript, including the disclosure of any use of AI.

No AI tools were used in the preparation of this manuscript.

### Author ORCIDs

C. Huu Nguyen https://orcid.org/0000-0003-2281-741X

N.V. Duong https://orcid.org/0009-0009-3943-5443

D.T. Bui https://orcid.org/0000-0003-3689-6200

H.T. Tran https://orcid.org/0009-0000-1331-3215

H.T. Bach https://orcid.org/0009-0008-9450-589X

Q.T. Phan https://orcid.org/0009-0008-4724-5466

V.K. Le https://orcid.org/0000-0003-4146-9836

D.T. Nguyen https://orcid.org/0009-0006-7164-1421

M.S. Nuraliev https://orcid.org/0000-0001-8291-2633

L.V. Averyanov https://orcid.org/0000-0001-8031-2925

C.W. Lin https://orcid.org/0000-0002-8564-5793

### Data availability

All of the data that support the findings of this study are available in the main text.
